# 2,3,4,6-Tetra-*O*-acetyl-1-*O*-(4-methoxy­cinnamo­yl)-β-d-glucopyran­ose

**DOI:** 10.1107/S1600536808004698

**Published:** 2008-02-22

**Authors:** Yuan-Yuan Liu, Shan Liu, Qing-Yan Chu, Hong-Jun Zhu

**Affiliations:** a Department of Applied Chemistry, College of Science, Nanjing University of Technology, Nanjing 210009, People’s Republic of China

## Abstract

Mol­ecules of the title compound, C_24_H_28_O_12_, are linked by inter­molecular C—H⋯O hydrogen bonds. Bond lengths and angles are normal.

## Related literature

For related literature, see: Loganathan & Trivedi (1987 ▶); Yu *et al.* (1991 ▶). For bond-length data, see: Allen *et al.* (1987 ▶).
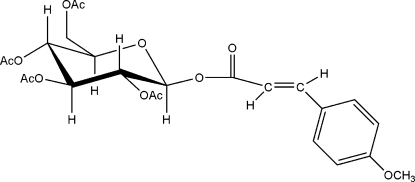


## Experimental

### 

#### Crystal data

C_24_H_28_O_12_
*M* *_r_* = 508.46
Monoclinic, 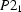

*a* = 9.972 (2) Å
*b* = 6.0580 (12) Å
*c* = 21.680 (4) Å
β = 97.19 (3)°
*V* = 1299.4 (4) Å^3^
*Z* = 2
Mo *K*α radiation
μ = 0.10 mm^−1^
*T* = 298 (2) K
0.40 × 0.10 × 0.10 mm

#### Data collection

Enraf–Nonius CAD-4 diffractometer
Absorption correction: ψ scan (North *et al.*, 1968 ▶) *T*_min_ = 0.959, *T*_max_ = 0.990
2955 measured reflections
2790 independent reflections
1578 reflections with *I* > 2σ(*I*)
*R*_int_ = 0.062
3 standard reflections every 200 reflections intensity decay: none

#### Refinement

*R*[*F* ^2^ > 2σ(*F* ^2^)] = 0.070
*wR*(*F* ^2^) = 0.189
*S* = 1.02
2790 reflections
319 parameters
1 restraint
H-atom parameters constrained
Δρ_max_ = 0.43 e Å^−3^
Δρ_min_ = −0.40 e Å^−3^

### 

Data collection: *CAD-4 Software* (Enraf–Nonius, 1985 ▶); cell refinement: *CAD-4 Software*; data reduction: *XCAD4* (Harms & Wocadlo, 1995 ▶); program(s) used to solve structure: *SHELXS97* (Sheldrick, 2008 ▶); program(s) used to refine structure: *SHELXL97* (Sheldrick, 2008 ▶); molecular graphics: *SHELXTL* (Sheldrick, 2008 ▶); software used to prepare material for publication: *SHELXTL*.

## Supplementary Material

Crystal structure: contains datablocks I, global. DOI: 10.1107/S1600536808004698/ez2118sup1.cif

Structure factors: contains datablocks I. DOI: 10.1107/S1600536808004698/ez2118Isup2.hkl

Additional supplementary materials:  crystallographic information; 3D view; checkCIF report

## Figures and Tables

**Table 1:** Hydrogen-bond geometry (Å, °)

*D*—H⋯*A*	*D*—H	H⋯*A*	*D*⋯*A*	*D*—H⋯*A*
C5—H5*A*⋯O3^i^	0.96	2.45	3.164 (12)	131
C10—H10*A*⋯O5^ii^	0.98	2.48	3.423 (9)	160
C13—H13*A*⋯O11^iii^	0.98	2.55	3.371 (8)	142
C24—H24*A*⋯O8^iv^	0.96	2.51	3.457 (10)	171

Symmetry codes: (i) 
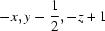
; (ii) 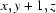; (iii) 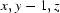; (iv) 
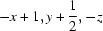
.

**Fig. 1.**
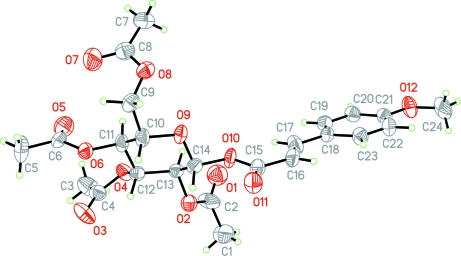
A drawing of the title molecular structure, with the atom-numbering scheme. Displacement ellipsoids are drawn at the 50% probability level. Hydrogen bonds are shown by dashed lines.

## supplementary crystallographic information

### Comment

1-*O*-(*p*-methoxycinnamoyl)-2,3,4,6-tetra-*O*-acetyl-β-*D*-glucopyranose is a type of glycolipid derivative (Loganathan *et al.*, 1987) that plays an important role in medical applications, such as anti-tumor and antibacterial applications. We report here the crystal structure of the title compound, (I).

The molecular structure of (I) is shown in Fig. 1. The bond lengths and angles are within normal ranges (Allen *et al.*, 1987).

In the crystal, molecules are linked to each other to form a three dimensional framework *via* intermolecular C—H···O hydrogen bonds.

### Experimental

The title compound, (I), was prepared by a method similar to that reported previously (Yu *et al.*, 1991). The crystals were obtained by dissolving compound I (1.5 g) in methanol (25 ml) and evaporating the solvent slowly at room temperature for about 10 d.

### Refinement

H atoms were positioned geometrically, with O—H = 0.82 and C—H = 0.93Å for aromatic H, and constrained to ride on their parent atoms, with *U*_iso_(H) = *xU*_eq_(C/O), where *x* = 1.2 for aromatic H and *x* = 1.5 for other H. In the absence of significant anomalous scattering effects 82 Friedel pairs have been merged.

### Crystal data

**Table:**

C_24_H_28_O_12_	*F*_000_ = 536
*M**_r_* = 508.46	*D*_x_ = 1.300 Mg m^−^^3^
Monoclinic, *P*2_1_	Mo *K*α radiation λ = 0.71073 Å
Hall symbol: P 2yb	Cell parameters from 25 reflections
*a* = 9.972 (2) Å	θ = 9–12º
*b* = 6.0580 (12) Å	µ = 0.11 mm^−^^1^
*c* = 21.680 (4) Å	*T* = 298 (2) K
β = 97.19 (3)º	Needle, colourless
*V* = 1299.4 (4) Å^3^	0.40 × 0.10 × 0.10 mm
*Z* = 2	

### Data collection

**Table:**

Enraf–Nonius CAD-4 diffractometer	*R*_int_ = 0.062
Radiation source: fine-focus sealed tube	θ_max_ = 26.0º
Monochromator: graphite	θ_min_ = 1.9º
*T* = 298(2) K	*h* = −11→11
ω/2θ scans	*k* = 0→7
Absorption correction: ψ scan(North *et al.*, 1968)	*l* = 0→26
*T*_min_ = 0.959, *T*_max_ = 0.990	3 standard reflections
2955 measured reflections	every 200 reflections
2790 independent reflections	intensity decay: none
1578 reflections with *I* > 2σ(*I*)	

### Refinement

**Table:**

Refinement on *F*^2^	Secondary atom site location: difference Fourier map
Least-squares matrix: full	Hydrogen site location: inferred from neighbouring sites
*R*[*F*^2^ > 2σ(*F*^2^)] = 0.070	H-atom parameters constrained
*wR*(*F*^2^) = 0.189	*w* = 1/[σ^2^(*F*_o_^2^) + (0.09*P*)^2^] where *P* = (*F*_o_^2^ + 2*F*_c_^2^)/3
*S* = 1.02	(Δ/σ)_max_ < 0.001
2790 reflections	Δρ_max_ = 0.43 e Å^−^^3^
319 parameters	Δρ_min_ = −0.40 e Å^−^^3^
1 restraint	Extinction correction: none
Primary atom site location: structure-invariant direct methods	

### Special details

**Table:**

Geometry. All e.s.d.'s (except the e.s.d. in the dihedral angle between two l.s. planes) are estimated using the full covariance matrix. The cell e.s.d.'s are taken into account individually in the estimation of e.s.d.'s in distances, angles and torsion angles; correlations between e.s.d.'s in cell parameters are only used when they are defined by crystal symmetry. An approximate (isotropic) treatment of cell e.s.d.'s is used for estimating e.s.d.'s involving l.s. planes.
Refinement. Refinement of *F*^2^ against ALL reflections. The weighted *R*-factor *wR* and goodness of fit *S* are based on *F*^2^, conventional *R*-factors *R* are based on *F*, with *F* set to zero for negative *F*^2^. The threshold expression of *F*^2^ > σ(*F*^2^) is used only for calculating *R*-factors(gt) *etc*. and is not relevant to the choice of reflections for refinement. *R*-factors based on *F*^2^ are statistically about twice as large as those based on *F*, and *R*- factors based on ALL data will be even larger.

### Fractional atomic coordinates and isotropic or equivalent isotropic displacement parameters (Å^2^)

**Table:**

	*x*	*y*	*z*	*U*_iso_*/*U*_eq_	
O1	0.5843 (6)	0.1875 (12)	0.2849 (3)	0.089	
C1	0.6777 (7)	0.468 (2)	0.3547 (4)	0.099 (3)	
H1A	0.7643	0.4270	0.3433	0.148*	
H1B	0.6804	0.4617	0.3990	0.148*	
H1C	0.6559	0.6153	0.3405	0.148*	
O2	0.4512 (4)	0.3681 (9)	0.3422 (2)	0.0591 (13)	
C2	0.5738 (7)	0.3138 (18)	0.3256 (4)	0.084 (3)	
O3	0.2760 (6)	0.0744 (11)	0.4807 (2)	0.0799 (17)	
C3	0.3974 (9)	−0.2530 (17)	0.4674 (4)	0.090 (3)	
H3A	0.3975	−0.2727	0.5113	0.135*	
H3B	0.4888	−0.2445	0.4580	0.135*	
H3C	0.3526	−0.3758	0.4457	0.135*	
O4	0.3228 (5)	−0.0182 (9)	0.38543 (19)	0.0567 (12)	
C4	0.3267 (7)	−0.0507 (14)	0.4480 (3)	0.0567 (18)	
O5	0.0717 (7)	−0.2870 (10)	0.3886 (3)	0.0846 (18)	
C5	−0.0937 (9)	−0.123 (2)	0.4448 (4)	0.116 (4)	
H5A	−0.1026	−0.2675	0.4619	0.174*	
H5B	−0.1795	−0.0758	0.4239	0.174*	
H5C	−0.0641	−0.0212	0.4776	0.174*	
O6	0.0213 (4)	0.0758 (8)	0.3744 (2)	0.0512 (12)	
C6	0.0086 (8)	−0.1292 (15)	0.3991 (3)	0.061 (2)	
O7	−0.1160 (7)	−0.2286 (11)	0.2512 (3)	0.095 (2)	
C7	−0.0606 (8)	−0.2633 (14)	0.1464 (3)	0.070 (2)	
H7A	−0.0739	−0.4181	0.1528	0.106*	
H7B	0.0305	−0.2382	0.1381	0.106*	
H7C	−0.1224	−0.2137	0.1116	0.106*	
O8	−0.0694 (5)	0.0730 (8)	0.1983 (2)	0.0547 (12)	
C8	−0.0852 (7)	−0.1400 (14)	0.2026 (4)	0.0600 (19)	
O9	0.1321 (4)	0.3455 (8)	0.24191 (19)	0.0492 (11)	
C9	−0.0883 (6)	0.2144 (13)	0.2493 (3)	0.0539 (18)	
H9A	−0.1431	0.1388	0.2767	0.065*	
H9B	−0.1368	0.3458	0.2337	0.065*	
O10	0.3339 (4)	0.4790 (8)	0.2226 (2)	0.0546 (12)	
C10	0.0445 (6)	0.2813 (12)	0.2859 (3)	0.0521 (18)	
H10A	0.0288	0.4098	0.3115	0.062*	
C11	0.1097 (6)	0.1011 (11)	0.3280 (3)	0.0482 (17)	
H11A	0.1150	−0.0366	0.3047	0.058*	
O11	0.2593 (5)	0.8310 (8)	0.2125 (2)	0.0652 (14)	
O12	0.8530 (5)	0.5383 (9)	−0.0491 (2)	0.0678 (15)	
C12	0.2491 (6)	0.1706 (11)	0.3578 (3)	0.0436 (15)	
H12A	0.2408	0.2826	0.3897	0.052*	
C13	0.3322 (6)	0.2602 (11)	0.3099 (3)	0.0455 (16)	
H13A	0.3588	0.1408	0.2835	0.055*	
C14	0.2546 (6)	0.4380 (12)	0.2704 (3)	0.0523 (18)	
H14A	0.2400	0.5710	0.2944	0.063*	
C15	0.3289 (7)	0.6851 (15)	0.1968 (3)	0.058 (2)	
C16	0.4170 (7)	0.7047 (14)	0.1476 (3)	0.0594 (19)	
H16A	0.4147	0.8349	0.1248	0.071*	
C17	0.4991 (7)	0.5466 (15)	0.1343 (3)	0.062 (2)	
H17A	0.4999	0.4189	0.1582	0.074*	
C18	0.5902 (6)	0.5500 (11)	0.0855 (3)	0.0448 (15)	
C19	0.6731 (7)	0.3730 (13)	0.0785 (3)	0.0558 (18)	
H19A	0.6720	0.2524	0.1049	0.067*	
C20	0.7586 (6)	0.3706 (13)	0.0325 (3)	0.0527 (17)	
H20A	0.8118	0.2474	0.0275	0.063*	
C21	0.7641 (6)	0.5531 (13)	−0.0059 (3)	0.0499 (17)	
C22	0.6811 (6)	0.7313 (13)	0.0001 (3)	0.0547 (18)	
H22A	0.6826	0.8520	−0.0263	0.066*	
C23	0.5952 (6)	0.7300 (13)	0.0457 (3)	0.0534 (18)	
H23A	0.5399	0.8513	0.0498	0.064*	
C24	0.8593 (8)	0.7190 (19)	−0.0897 (4)	0.088 (3)	
H24A	0.9251	0.6896	−0.1174	0.132*	
H24B	0.8848	0.8495	−0.0659	0.132*	
H24C	0.7723	0.7410	−0.1134	0.132*	

### Atomic displacement parameters (Å^2^)

**Table:**

	*U*^11^	*U*^22^	*U*^33^	*U*^12^	*U*^13^	*U*^23^
O1	0.089	0.089	0.089	0.000	0.011	0.000
C1	0.062 (5)	0.131 (8)	0.109 (7)	−0.030 (6)	0.033 (5)	−0.042 (7)
O2	0.049 (3)	0.071 (3)	0.061 (3)	−0.011 (3)	0.019 (2)	−0.012 (3)
C2	0.051 (4)	0.126 (8)	0.079 (5)	−0.008 (5)	0.027 (4)	−0.054 (6)
O3	0.088 (4)	0.104 (5)	0.049 (3)	0.037 (4)	0.014 (3)	−0.006 (3)
C3	0.113 (7)	0.090 (7)	0.066 (5)	0.025 (7)	0.006 (5)	−0.001 (5)
O4	0.067 (3)	0.060 (3)	0.046 (3)	0.009 (3)	0.017 (2)	−0.005 (3)
C4	0.055 (4)	0.060 (5)	0.055 (4)	0.001 (4)	0.007 (3)	−0.009 (4)
O5	0.119 (5)	0.050 (4)	0.086 (4)	−0.014 (4)	0.016 (3)	0.008 (3)
C5	0.118 (8)	0.143 (10)	0.095 (7)	−0.048 (8)	0.050 (6)	0.023 (7)
O6	0.051 (3)	0.052 (3)	0.054 (3)	−0.008 (2)	0.021 (2)	−0.010 (2)
C6	0.075 (5)	0.052 (5)	0.058 (4)	−0.030 (5)	0.011 (4)	0.001 (4)
O7	0.132 (5)	0.074 (4)	0.084 (4)	−0.025 (4)	0.033 (4)	0.005 (4)
C7	0.077 (5)	0.069 (6)	0.061 (4)	−0.003 (5)	−0.006 (4)	−0.010 (5)
O8	0.056 (3)	0.046 (3)	0.061 (3)	−0.005 (2)	0.004 (2)	0.000 (3)
C8	0.052 (4)	0.059 (5)	0.068 (5)	0.002 (4)	0.003 (4)	−0.005 (5)
O9	0.044 (2)	0.049 (3)	0.058 (3)	−0.005 (2)	0.020 (2)	0.009 (2)
C9	0.042 (4)	0.051 (4)	0.070 (5)	0.000 (4)	0.011 (3)	0.000 (4)
O10	0.060 (3)	0.042 (2)	0.069 (3)	−0.009 (2)	0.038 (2)	0.006 (3)
C10	0.046 (4)	0.058 (5)	0.057 (4)	−0.002 (3)	0.023 (3)	0.001 (4)
C11	0.054 (4)	0.042 (4)	0.054 (4)	0.001 (3)	0.029 (3)	0.000 (3)
O11	0.086 (4)	0.044 (3)	0.073 (3)	0.012 (3)	0.037 (3)	−0.003 (3)
O12	0.073 (3)	0.074 (4)	0.063 (3)	0.023 (3)	0.033 (3)	0.015 (3)
C12	0.050 (4)	0.033 (3)	0.048 (3)	0.002 (3)	0.010 (3)	−0.005 (3)
C13	0.048 (4)	0.043 (4)	0.046 (3)	−0.006 (3)	0.012 (3)	−0.011 (3)
C14	0.054 (4)	0.047 (4)	0.061 (4)	−0.011 (3)	0.028 (3)	−0.021 (4)
C15	0.049 (4)	0.073 (5)	0.056 (4)	−0.018 (4)	0.026 (3)	−0.010 (4)
C16	0.061 (4)	0.063 (5)	0.059 (4)	−0.016 (4)	0.025 (3)	0.002 (4)
C17	0.052 (4)	0.089 (6)	0.047 (4)	0.001 (4)	0.013 (3)	0.005 (4)
C18	0.042 (3)	0.049 (4)	0.045 (3)	−0.006 (3)	0.012 (3)	0.002 (3)
C19	0.055 (4)	0.060 (5)	0.051 (4)	−0.006 (4)	0.003 (3)	0.003 (4)
C20	0.048 (4)	0.058 (4)	0.053 (4)	0.004 (4)	0.013 (3)	−0.004 (4)
C21	0.044 (3)	0.062 (4)	0.045 (4)	0.002 (4)	0.012 (3)	−0.005 (4)
C22	0.059 (4)	0.053 (4)	0.054 (4)	0.002 (4)	0.016 (3)	0.010 (4)
C23	0.043 (4)	0.066 (5)	0.054 (4)	0.001 (4)	0.015 (3)	0.001 (4)
C24	0.083 (5)	0.127 (8)	0.064 (5)	−0.001 (7)	0.048 (4)	−0.002 (6)

### Geometric parameters (Å, °)

**Table:**

O1—C2	1.183 (9)	O10—C15	1.366 (9)
C1—C2	1.475 (12)	O10—C14	1.403 (7)
C1—H1A	0.9600	C10—C11	1.516 (9)
C1—H1B	0.9600	C10—H10A	0.9800
C1—H1C	0.9600	C11—C12	1.518 (9)
O2—C2	1.358 (8)	C11—H11A	0.9800
O2—C13	1.456 (7)	O11—C15	1.199 (8)
O3—C4	1.193 (8)	O12—C21	1.372 (7)
C3—C4	1.450 (12)	O12—C24	1.411 (11)
C3—H3A	0.9600	C12—C13	1.509 (8)
C3—H3B	0.9600	C12—H12A	0.9800
C3—H3C	0.9600	C13—C14	1.525 (9)
O4—C4	1.367 (8)	C13—H13A	0.9800
O4—C12	1.448 (8)	C14—H14A	0.9800
O5—C6	1.182 (10)	C15—C16	1.469 (8)
C5—C6	1.509 (10)	C16—C17	1.315 (10)
C5—H5A	0.9600	C16—H16A	0.9300
C5—H5B	0.9600	C17—C18	1.479 (8)
C5—H5C	0.9600	C17—H17A	0.9300
O6—C6	1.365 (9)	C18—C19	1.375 (9)
O6—C11	1.425 (7)	C18—C23	1.396 (9)
O7—C8	1.253 (9)	C19—C20	1.390 (8)
C7—C8	1.477 (10)	C19—H19A	0.9300
C7—H7A	0.9600	C20—C21	1.389 (10)
C7—H7B	0.9600	C20—H20A	0.9300
C7—H7C	0.9600	C21—C22	1.376 (9)
O8—C8	1.305 (9)	C22—C23	1.386 (8)
O8—C9	1.431 (8)	C22—H22A	0.9300
O9—C14	1.413 (7)	C23—H23A	0.9300
O9—C10	1.426 (7)	C24—H24A	0.9600
C9—C10	1.512 (9)	C24—H24B	0.9600
C9—H9A	0.9700	C24—H24C	0.9600
C9—H9B	0.9700		
			
C2—C1—H1A	109.5	C10—C11—C12	110.9 (5)
C2—C1—H1B	109.5	O6—C11—H11A	110.5
H1A—C1—H1B	109.5	C10—C11—H11A	110.5
C2—C1—H1C	109.5	C12—C11—H11A	110.5
H1A—C1—H1C	109.5	C21—O12—C24	117.3 (6)
H1B—C1—H1C	109.5	O4—C12—C13	106.0 (5)
C2—O2—C13	118.1 (5)	O4—C12—C11	110.3 (5)
O1—C2—O2	121.4 (7)	C13—C12—C11	111.0 (5)
O1—C2—C1	127.2 (7)	O4—C12—H12A	109.8
O2—C2—C1	109.9 (7)	C13—C12—H12A	109.8
C4—C3—H3A	109.5	C11—C12—H12A	109.8
C4—C3—H3B	109.5	O2—C13—C12	108.3 (5)
H3A—C3—H3B	109.5	O2—C13—C14	106.2 (5)
C4—C3—H3C	109.5	C12—C13—C14	110.9 (5)
H3A—C3—H3C	109.5	O2—C13—H13A	110.4
H3B—C3—H3C	109.5	C12—C13—H13A	110.4
C4—O4—C12	118.3 (5)	C14—C13—H13A	110.4
O3—C4—O4	122.5 (7)	O10—C14—O9	106.4 (5)
O3—C4—C3	126.3 (7)	O10—C14—C13	104.3 (5)
O4—C4—C3	111.2 (7)	O9—C14—C13	108.4 (5)
C6—C5—H5A	109.5	O10—C14—H14A	112.4
C6—C5—H5B	109.5	O9—C14—H14A	112.4
H5A—C5—H5B	109.5	C13—C14—H14A	112.4
C6—C5—H5C	109.5	O11—C15—O10	123.4 (5)
H5A—C5—H5C	109.5	O11—C15—C16	124.5 (7)
H5B—C5—H5C	109.5	O10—C15—C16	112.1 (7)
C6—O6—C11	118.1 (5)	C17—C16—C15	123.1 (7)
O5—C6—O6	125.7 (6)	C17—C16—H16A	118.5
O5—C6—C5	124.5 (9)	C15—C16—H16A	118.5
O6—C6—C5	109.8 (8)	C16—C17—C18	127.0 (7)
C8—C7—H7A	109.5	C16—C17—H17A	116.5
C8—C7—H7B	109.5	C18—C17—H17A	116.5
H7A—C7—H7B	109.5	C19—C18—C23	118.2 (6)
C8—C7—H7C	109.5	C19—C18—C17	120.1 (7)
H7A—C7—H7C	109.5	C23—C18—C17	121.8 (6)
H7B—C7—H7C	109.5	C18—C19—C20	121.3 (7)
C8—O8—C9	120.5 (6)	C18—C19—H19A	119.4
O7—C8—O8	121.9 (7)	C20—C19—H19A	119.4
O7—C8—C7	124.1 (8)	C19—C20—C21	119.6 (7)
O8—C8—C7	113.9 (7)	C19—C20—H20A	120.2
C14—O9—C10	112.6 (5)	C21—C20—H20A	120.2
O8—C9—C10	112.0 (5)	O12—C21—C22	124.3 (6)
O8—C9—H9A	109.2	O12—C21—C20	115.7 (6)
C10—C9—H9A	109.2	C22—C21—C20	119.9 (6)
O8—C9—H9B	109.2	C21—C22—C23	119.7 (7)
C10—C9—H9B	109.2	C21—C22—H22A	120.2
H9A—C9—H9B	107.9	C23—C22—H22A	120.2
C15—O10—C14	117.9 (5)	C22—C23—C18	121.3 (7)
O9—C10—C9	107.0 (5)	C22—C23—H23A	119.4
O9—C10—C11	110.3 (5)	C18—C23—H23A	119.4
C9—C10—C11	113.8 (6)	O12—C24—H24A	109.5
O9—C10—H10A	108.5	O12—C24—H24B	109.5
C9—C10—H10A	108.5	H24A—C24—H24B	109.5
C11—C10—H10A	108.5	O12—C24—H24C	109.5
O6—C11—C10	104.2 (5)	H24A—C24—H24C	109.5
O6—C11—C12	110.1 (5)	H24B—C24—H24C	109.5
			
C13—O2—C2—O1	2.2 (13)	O4—C12—C13—C14	171.1 (5)
C13—O2—C2—C1	169.5 (7)	C11—C12—C13—C14	51.3 (7)
C12—O4—C4—O3	−2.7 (10)	C15—O10—C14—O9	94.4 (7)
C12—O4—C4—C3	176.6 (6)	C15—O10—C14—C13	−151.1 (5)
C11—O6—C6—O5	5.5 (11)	C10—O9—C14—O10	176.0 (5)
C11—O6—C6—C5	−177.4 (6)	C10—O9—C14—C13	64.4 (6)
C9—O8—C8—O7	−0.6 (11)	O2—C13—C14—O10	71.6 (5)
C9—O8—C8—C7	−179.3 (5)	C12—C13—C14—O10	−170.9 (5)
C8—O8—C9—C10	100.3 (8)	O2—C13—C14—O9	−175.3 (4)
C14—O9—C10—C9	172.6 (6)	C12—C13—C14—O9	−57.8 (6)
C14—O9—C10—C11	−63.1 (7)	C14—O10—C15—O11	−0.9 (10)
O8—C9—C10—O9	46.1 (8)	C14—O10—C15—C16	179.8 (6)
O8—C9—C10—C11	−76.0 (7)	O11—C15—C16—C17	175.3 (7)
C6—O6—C11—C10	147.6 (6)	O10—C15—C16—C17	−5.5 (10)
C6—O6—C11—C12	−93.3 (7)	C15—C16—C17—C18	179.4 (7)
O9—C10—C11—O6	172.1 (5)	C16—C17—C18—C19	178.2 (7)
C9—C10—C11—O6	−67.7 (7)	C16—C17—C18—C23	−1.7 (11)
O9—C10—C11—C12	53.6 (7)	C23—C18—C19—C20	−0.8 (10)
C9—C10—C11—C12	173.9 (5)	C17—C18—C19—C20	179.2 (6)
C4—O4—C12—C13	140.7 (6)	C18—C19—C20—C21	2.1 (10)
C4—O4—C12—C11	−99.0 (6)	C24—O12—C21—C22	0.3 (10)
O6—C11—C12—O4	78.9 (6)	C24—O12—C21—C20	178.8 (7)
C10—C11—C12—O4	−166.2 (5)	C19—C20—C21—O12	178.8 (6)
O6—C11—C12—C13	−163.9 (5)	C19—C20—C21—C22	−2.7 (10)
C10—C11—C12—C13	−49.0 (7)	O12—C21—C22—C23	−179.7 (6)
C2—O2—C13—C12	129.9 (7)	C20—C21—C22—C23	1.9 (10)
C2—O2—C13—C14	−110.9 (7)	C21—C22—C23—C18	−0.6 (10)
O4—C12—C13—O2	−72.7 (6)	C19—C18—C23—C22	0.1 (10)
C11—C12—C13—O2	167.5 (5)	C17—C18—C23—C22	180.0 (6)

### Hydrogen-bond geometry (Å, °)

**Table:**

*D*—H···*A*	*D*—H	H···*A*	*D*···*A*	*D*—H···*A*
C5—H5A···O3^i^	0.96	2.45	3.164 (12)	131
C10—H10A···O5^ii^	0.98	2.48	3.423 (9)	160
C13—H13A···O11^iii^	0.98	2.55	3.371 (8)	142
C24—H24A···O8^iv^	0.96	2.51	3.457 (10)	171

Symmetry codes: (i) −*x*, *y*−1/2, −*z*+1; (ii) *x*, *y*+1, *z*; (iii) *x*, *y*−1, *z*; (iv) −*x*+1, *y*+1/2, −*z*.
